# Measuring Global Dietary Diversity by Considering Nutritional Functional Dissimilarity and Dietary Guidelines

**DOI:** 10.3390/foods14101759

**Published:** 2025-05-15

**Authors:** Shiwen Quan, Wenbo Zhu

**Affiliations:** Rural Development Institute, Chinese Academy of Social Sciences, Beijing 100732, China; quanshw@cass.org.cn

**Keywords:** dietary diversity, nutrition functional dissimilarity, dietary guidelines, dietary pattern, healthy diets, food consumption

## Abstract

Dietary diversity is essential for healthy diets and crucial for academic research and policymaking. However, existing measures often lack conceptual clarity, which limits their interpretability. This study proposes a new framework that classifies dietary diversity indices along two dimensions: whether they account for nutritional functional dissimilarity and whether they incorporate dietary guidelines. Based on this framework, four index types are defined. Using per capita consumption data for 14 food categories across countries from 1981 to 2022, eight indices were applied to assess global dietary diversity and its variation across 13 dietary patterns. The results show a general upward trend in global dietary diversity and dietary quality, with notable regional disparities influenced by dietary patterns, resources, culture, and socioeconomic factors. This study also finds non-linear links between dietary diversity, income, and urbanization, consistent with Bennett’s Law and empirical evidence. These findings underscore the utility of the proposed indices in capturing complex dietary dynamics. This study recommends context-specific use of indices, policy attention in developing countries to maintain diversity during dietary transitions, and the development of more inclusive dietary guidelines that emphasize not only variety but also balance and nutritional function.

## 1. Introduction

Dietary diversity is essential for optimal nutrition. The human body requires a complex array of nutrients, including carbohydrates, fats, proteins, vitamins, minerals, and dietary fibers, which cannot be adequately supplied by a single food source. Consequently, humans seek varied diets to satisfy both nutritional requirements and gustatory preferences [1]. In the context of globalization, however, dietary patterns are becoming increasingly homogenized, leading to nutritional imbalances and a rise in non-communicable chronic diseases [2]. This trend has prompted increasing attention to dietary diversity in both research and practice [3,4].

The global interconnectedness of food supply and consumption networks necessitates a systematic understanding of dietary diversity across nations. Such knowledge can elucidate regional differences in dietary structures and nutritional statuses, inform the development of comprehensive public health policies to achieve nutritional goals, and ultimately improve human welfare. Moreover, dietary diversity is intrinsically linked to food security; countries that are heavily reliant on a limited range of food sources are more vulnerable to food security risks [3,5]. Thus, measuring and comparing dietary diversity across countries provides valuable insights into national food security vulnerabilities.

The primary challenge in studying global dietary diversity lies in obtaining comprehensive food consumption data. While individual-level dietary surveys would theoretically provide the most accurate measurements [6,7,8], the logistical challenges of conducting such surveys on a global scale with consistent methodologies render this approach impractical for large-scale studies. Currently, macro-level food consumption data offer the most viable means of generating comparable global dietary diversity results. For instance, Krivonos and Kuhn [9] and Miyamoto et al. [10] utilized food balance data from the Food and Agriculture Organization (FAO) to measure dietary diversity across multiple countries.

Another significant challenge is the lack of a universally accepted method for measuring dietary diversity, which hinders the comparison of conclusions drawn from existing empirical studies. Current research broadly employs three types of indicators: (1) count-based indicators, such as the Dietary Diversity Score (DDS) and Food Variety Score (FVS), which are primarily used with micro-level dietary data [6,11]; (2) indices that consider both the variety and distribution of food consumption, such as the Shannon Entropy Index and the Simpson Index, which are applicable to both micro and macro data [12,13]; and (3) health-oriented dietary diversity indices, which are constructed using dietary guideline information, either through scoring methods based on reference intake levels [14,15] or by adjusting existing indices to create health-focused dietary diversity indices (HFD) [16,17].

This inconsistency in indicators reveals underlying divergences in the conceptualization of dietary diversity. Currently, there is no universally accepted definition [18]. Count-based indicators focus solely on the number of food types consumed, disregarding quantity variations. Distribution indices account for both variety and quantity but overlook functional differences among food types. Indices based on dietary guidelines can more intuitively reflect diet healthfulness but often conflate dietary diversity with dietary quality. For instance, Marshall et al. [19] noted significant disagreement in labeling similarly constructed indices as measures of *diversity* or *quality*.

Despite progress in the field, the varying scales of micro-level food consumption data and inconsistent measurement methods have hindered comprehensive research. Existing studies employ disparate data and methods to measure dietary diversity within individual countries or regions, which precludes systematic cross-national comparisons. Furthermore, the relationship between dietary diversity and dietary quality remains poorly understood.

This paper makes two primary contributions to the field. First, we propose a new comprehensive measurement system based on the concept and connotation of dietary diversity. This system reclassifies existing dietary diversity indices and introduces novel indices to address current methodological shortcomings. Second, utilizing macro food consumption data published by the FAO, we measure global dietary diversity using the newly developed indices, providing comparable results across dietary patterns and time periods. These findings will serve as a valuable reference for the formulation of global nutritional and health policies.

## 2. Methods

### 2.1. Concept and Measurement Principles of Dietary Diversity

The concept of dietary diversity has its roots in biodiversity. The Convention on Biological Diversity defines biodiversity as “variability among living organisms from all sources including, inter alia, terrestrial, marine and other aquatic ecosystems and the ecological complexes of which they are part; this includes diversity within species, between species and of ecosystems” (The Convention on Biological Diversity: https://www.cbd.int/convention/articles/default.shtml?a=cbd-02, accessed on 12 December 2024). FAO further contextualizes biodiversity within agriculture and food systems, encompassing “the variety and variability of animals, plants, and microorganisms at the genetic, species, and ecosystem levels that sustain the ecosystem structures, functions, and processes in and around production systems, and that provide food and nonfood agricultural products”. As the concept of diversity transitioned into food consumption research, the focus shifted. While biodiversity typically emphasizes species richness, dietary diversity concentrates more on categorizing food groups with similar nutritional functions rather than on the distribution of individual food types [20,21].

The measurement of dietary diversity largely draws from ecological approaches to measuring biodiversity. However, like biodiversity, dietary diversity lacks a universally accepted, quantifiable definition. In practice, the interpretation of diversity often depends on the indices chosen or constructed by researchers. Hanley-Cook et al. [21] explicitly introduced three fundamental components of biodiversity measurement into dietary diversity studies: richness, evenness, and disparity. *Richness* refers to the absolute number of food types in the dietary composition, typically measured by counting. *Evenness* denotes the degree to which the proportions of various foods in the total dietary intake are uniform, commonly measured using the Shannon Entropy Index and the Simpson Index. *Disparity* reflects the degree of nutritional functional dissimilarities between food types in the dietary composition, with greater functional dissimilarities indicating higher disparity.

In biodiversity research, disparity is primarily measured using distance-based or tree-based methods [22]. Current dietary diversity research predominantly examines richness and evenness, with only a few studies addressing disparity [23]. Diversity indices that only consider richness or evenness, termed *species-neutral indices* by Hanley-Cook et al. [21], implicitly assume that different food species are nutritionally independent. In contrast, indices that account for disparity are referred to as *functional dissimilarity indices* or *similarity-sensitive indices*. Constructing these indices requires information on both food consumption amounts or proportions and the nutritional functions of various foods.

A key distinction between dietary diversity and biodiversity lies in the existence of normative standards. Although there is a close relationship between biodiversity and ecosystem function, existing research does not seem to confirm the existence of a normative biodiversity standard for achieving optimal ecosystem function. Due to the influence of natural geographical conditions, there are often significant differences in biodiversity among different ecosystems. Conversely, nutritional and medical evidence supports the existence of normative standards for food intake to maintain and promote human health. Consequently, the scientific validity of recommended food intakes in dietary guidelines is widely recognized. Therefore, dietary diversity indices can be constructed not only based on actual dietary conditions but also by considering the gap between actual intake and the recommended standards in dietary guidelines.

From a conceptual perspective, indices constructed solely on actual food intake may not necessarily reflect a healthier diet, even if they indicate high richness, evenness, and disparity. When reference intakes are used as benchmarks for dietary quality, a smaller gap between actual dietary conditions and reference standards indicates higher dietary quality or a healthier diet. This logic underpins the construction of *dietary guideline-based indices*. The distinction between these indices and traditional diversity measures reflects the nuanced relationship between dietary diversity and dietary quality. While empirical research has generally confirmed a positive correlation between dietary diversity and individual health, logically, higher dietary diversity does not necessarily equate to higher dietary quality; their relationship is not strictly monotonically increasing. Dietary diversity serves as an empirical indicator of dietary quality but is not conceptually a subset of it. This may explain why some empirical studies [24] have failed to confirm that dietary diversity promotes human health. In contrast, dietary guideline-based diversity can be understood as a subset of dietary quality, measuring dietary quality from a diversity perspective.

However, there is no consensus on how to construct *dietary guideline-based indices*. The existing literature presents two main approaches: scoring methods that assign scores to actual food intake or ranges based on reference intakes [14,15] and the modification of dietary diversity indices by incorporating dietary guideline information, such as Drescher et al.’s [16] Healthy Food Diversity (HFD) Index, which adds a health value multiplier to the Simpson Index. Indices derived from scoring methods essentially measure dietary quality, and their construction is not directly related to the three components of the diversity concept. Although the HFD Index maintains a connection with the concept of diversity, the form of the health value multiplier and its method of introduction are highly subjective, and the mathematical properties of the constructed indices remain unclear.

### 2.2. A New Framework for Dietary Diversity Indices

Based on the earlier discussion, we classify dietary diversity indices into four categories, depending on whether they consider functional disparity and incorporate dietary guidelines: species-neutral indices, functional dissimilarity indices, dietary guideline-based species-neutral indices, and dietary guideline-based functional dissimilarity indices. Since richness indices, represented by count-based indicators, can be derived or transformed from evenness indices and are not suitable for evaluating macro-level data, this paper will not consider the relevant indices (Table 1).

The concept of disparity is crucial in dietary diversity measurement. As illustrated by specific cases, under given conditions of richness and evenness, a dietary structure comprising pork, beef, and poultry exhibits lower diversity than one comprising pork, beef, and rice, as the latter combination offers a broader range of nutritional functions. This difference also explains why indices (1) and (3) fundamentally diverge from indices (2) and (4). Furthermore, assuming dietary guidelines prescribe healthy diets that include grains, meats, and vegetables, both aforementioned dietary patterns would exhibit lower diversity compared to a pattern consisting of pork, lettuce, and rice. The latter configuration aligns more closely with recommended dietary standards. This distinction also differentiates indices (1) and (2) from indices (3) and (4), as the dietary guideline-based indices explicitly account for adherence to nutritional recommendations in diversity quantification.

#### 2.2.1. Species-Neutral Indices

Species-neutral indices account solely for distribution evenness, disregarding functional disparity, and are thus often referred to as *evenness* indices. Common examples include the Shannon Entropy Index and the Simpson Index, which are represented in Equations (1) and (2), respectively, as follows:

(1) $$\mathrm{EI}=-\sum_{i}^{S}p_{i}\ln\left(p_{i}\right)$$

(2) $$\mathrm{BI}=1-\sum_{i}^{S}p_{i}^{2}$$

where the subscript i∈S represents food species, and $p_{i}$ denotes the proportion of food species, *i*, in total intake. The general form of evenness indices can be summarized as the expected value of $f\left(p_{i}\right)$, which is expressed as $\sum p_{i}f\left(p_{i}\right)$, where the function $f\left(p_{i}\right)$ defines the rarity of the food species, i [25,26]. Essentially, species-neutral indices measure the average rarity of foods in a diet. An increase in the Shannon Entropy Index (*EI*) and Simpson Index (*BI*) indicates a higher average rarity.

At the individual food level, a higher proportion of a food implies lower rarity, i.e., $\partial f\left(p_{i}\right)/\partial p_{i}<0$. Thus, it follows that in the Shannon Entropy Index, $f\left(p_{i}\right)=-\ln\left(p_{i}\right)$, and in the Simpson Index, $f\left(p_{i}\right)=1-p_{i}$, the differences in these functional forms reflect the varying sensitivities of these indices to rare and abundant foods.

#### 2.2.2. Functional Dissimilarity Indices

The functional dissimilarity index considers both distribution *evenness* and functional *disparity*. In ecology, the most widely used index is the *Q* index, proposed by Rao [27,28] and also known as the *quadratic entropy index*. More recently, Green et al. [29] and Wang et al. [30] introduced the *Q* index into the field of food consumption to measure dietary diversity. It is expressed in Equation (3) as follows:

(3) $$Q_{B}=\sum_{i}^{S}\sum_{j}^{S}d_{ij}p_{i}p_{j}$$

where $p_{i}$ and $p_{j}$ represent the proportions of food species *i* and *j*, respectively, in total food intake, and $d_{ij}$ denotes the dissimilarity in nutritional function between species *i* and *j.* Dissimilarity is typically measured using distance metrics such as Manhattan, Euclidean, or cosine distances, satisfying $d_{ij}=d_{ji}$ and $d_{ii}=0$.

The *Q* index is related to the Simpson Index [25]. Without considering nutritional functions, the rarity of food species *i* is $p_{i}=1-\sum_{j\neq i}^{S}p_{j}$. However, when nutritional functions are included, rarity also depends on the nutritional differences between species. If the nutritional function of species *i* is similar to that of others, its rarity will not be high, even with a low consumption share. Ricotta and Szeidl [25] modified $f\left(p_{i}\right)$ to $f\left(1-\sum_{j\neq i}^{S}d_{ij}p_{j}\right)$, thus incorporating nutritional dissimilarity, where $\sum_{j\neq i}^{S}d_{ij}p_{j}$ represents the average difference in nutritional function between food *i* and all other food species. Accordingly, the Simpson Index can be rewritten as $BI=\sum_{i}^{S}p_{i}\left[1-\left(1-\sum_{j\neq i}^{S}d_{ij}p_{j}\right)\right]$.

By substituting the rarity function, $f\left(1-\sum_{j\neq i}^{S}d_{ij}p_{j}\right)$, into the Shannon Entropy Index (*EI*), Ricotta and Szeidl [25] further derived the *Q_E_* index as follows:

(4) $$Q_{E}=-\sum_{i}^{S}p_{i}\ln\left(1-\sum_{j\neq i}^{S}d_{ij}p_{j}\right)$$

Both the *Q_E_* and *Q_B_* indices measure the weighted average rarity of foods based on their nutritional functions. The larger the index, the higher the weighted average rarity. If nutritional neutrality is assumed, i.e., *d_ij_* = 1, ∀i≠j and *d_ii_* = 0, the *Q_E_* and *Q_B_* indices reduce to *EI* and *BI* indices, respectively. While the *Q_E_* index has not yet been widely used in empirical dietary studies, it provides a more nuanced approach to assessing diversity by considering nutritional functions. Compared to *Q_E_* index, the functional form of *Q_B_* index is more concise and does not require additional constraints on the distance function. However, the calculation of the *Q_E_* index requires that *d_ij_* ≤ 1.

#### 2.2.3. Dietary Guideline-Based Species-Neutral Indices

The third type of index incorporates dietary guidelines to assess the deviation between actual diets and recommended standards. These indices, referred to as dietary guideline-based species-neutral indices, measure the evenness discrepancy between actual diets and reference standards using statistical distance measures. Traditional distance metrics like Manhattan or Euclidean distance are inadequate for this purpose. Instead, we introduce the Kullback–Leibler (KL) divergence from information theory, also known as Relative Entropy, which is represented as the *D_E_* index in Equation (5) as follows:

(5) $$D_{E}=\sum_{i}^{S}p_{i}\left(\ln\left(p_{i}\right)-\ln\left(q_{i}\right)\right)$$

where $q_{i}$ represents the proportion of the recommended intake of food species *i* based on dietary guidelines, while $p_{i}$ represents the actual intake. The KL divergence measures the distance between two probability distributions, where *D_E_* ≥ 0 according to Gibbs’ inequality. A larger *D_E_* index indicates lower diet quality and less healthy eating patterns. This paper also introduces the *D_B_* index based on the Simpson Index. To satisfy the condition *D_B_* ≥ 0, the absolute value of the difference between the actual and reference intake proportions is taken when constructing the *D_B_* index, as follows:

(6) $$D_{B}=\sum_{i}^{S}p_{i}\left|p_{i}-q_{i}\right|$$

Both *D_B_* and *D_E_* indices reflect the evenness gap between the actual and reference diets, with larger indices indicating less healthy diets. It is important to note that while KL divergence does not satisfy symmetry, this is not required for comparative analysis when using a unified dietary guideline as the reference standard.

#### 2.2.4. Dietary Guideline-Based Functional Dissimilarity Indices

The fourth type of index combines functional dissimilarity with dietary guidelines, referred to as the *DQ* index. This index measures the functional dissimilarity between actual diets and reference diets.

Building on the concept of rarity functions, we express the general form of the cross-entropy function as $\sum p_{i}f\left(q_{i}\right)$, where the function $f\left(q_{i}\right)$ defines the rarity of food *i* under the normative standard. Cross-entropy reflects the average rarity of foods in a diet relative to a normative standard, and the difference between cross-entropy and Shannon entropy reflects the gap between actual and reference diets. After adjusting for nutritional functions like Ricotta and Szeidl [25], we modify $f\left(q_{i}\right)$ to $f\left(1-\sum_{j\neq i}^{S}d_{ij}q_{j}\right)$, thus accounting for dissimilarity, where $\sum_{j\neq i}^{S}d_{ij}q_{j}$ represents the average difference in nutritional function between species *i* and all other foods in the reference dietary composition. Accordingly, by substituting two rarity functions into the *D_E_* and *D_B_* indices, we can derive the *DQ_E_* and *DQ_B_* indices. These indices do not satisfy the symmetry condition either, as discussed in the third type of index as follows:

(7) $$DQ_{E}=\sum_{i}^{S}p_{i}\left|\ln\left(1-\sum_{j\neq i}^{S}d_{ij}p_{j}\right)-\ln\left(1-\sum_{j\neq i}^{S}d_{ij}q_{j}\right)\right|$$

(8) $$DQ_{B}=\sum_{i}^{S}\sum_{j}^{S}d_{ij}p_{i}\left|p_{j}-q_{j}\right|$$

The *DQ_E_* index requires an additional constraint of *d_ij_* ≤ 1. Since negative values are difficult to interpret, we use the absolute value of the differences. The *DQ_E_* and *DQ_B_* indices measure the weighted average nutritional dissimilarity between actual and reference diets, with larger values indicating greater gaps and lower dietary health.

It is important to note that the value ranges of the four types of indices differ. Additionally, the first two indices are positive, while the latter two are negative. To facilitate comparison, the four types of indices were first normalized within the sample range using min–max standardization, restricting their values to the range of [0, 1]. Subsequently, the negative indices were converted into positive indices through a complement transformation method.

## 3. Materials

### 3.1. Food Consumption Data

This study examines the dietary diversity of the global population by calculating four types of dietary diversity indices. The purpose is to explore the relationships between these indices and analyze the representative characteristics of global dietary diversity. The primary data on food consumption are sourced from the Food Balance Sheets (FBS) compiled by the Food and Agriculture Organization (FAO) of the United Nations. These data provide per capita food consumption figures for various countries and regions worldwide. It should be noted that the FBS measure of “food available for consumption per capita” was used. Conceptually, this measure reflects food availability, defined as the total quantity of food accessible to a country’s residents through retail markets for human consumption, including losses and waste at the retail and consumption stages. As such, “per capita food consumption” may overestimate the actual intake by residents. However, since the dietary diversity indices in this study are based solely on the proportional composition of food consumption categories rather than absolute consumption values, any potential overestimation, assuming it is systemic rather than structural, has minimal impact on the results.

Over the years, the FAO has made multiple updates to the FBS, including revisions to its underlying data, compilation methods, and food classifications. These updates have created discrepancies between older and newer datasets, resulting in structural breaks in global food consumption data, particularly between 2010 and 2013, due to adjustments in statistical methodologies. To address this issue, the raw FBS data were adjusted in three steps. First, structural breaks were eliminated. The year 2010 was selected as the breakpoint, with post-2010 data retained to ensure the continuity of future datasets. For years prior to 2010, the ratio of per capita consumption levels between earlier years and the 2010 level in the old dataset was calculated, and this ratio was assumed to remain constant in the new dataset. These ratios were then used to reconstruct historical data under the new standards, resulting in a dataset of per capita food consumption that is comparable over time. Second, food categories were standardized. Following the classification standards of the new dataset, food codes and names were harmonized before and after the adjustment, with miscellaneous and non-food categories removed. To ensure consistency with healthy dietary standards, the raw data were consolidated into 14 major food categories: cereals, roots and tubers, pulses, oils and fats, pork, beef and lamb, poultry, eggs, dairy, seafood, vegetables, fruits, nuts, and sugar. Finally, missing values were imputed. Intermediate missing values were interpolated, while values at the beginning or end of the dataset were filled using the grouped average method, which considers time, geography, and income groupings. Priority was given to mean values from countries within the same time period, region, and income group.

Following these adjustments, this study generated annual per capita consumption data for 14 food categories across major countries and regions globally. The sample period for calculating dietary diversity indices spans from 1981 to 2022. Additionally, to analyze the association between dietary diversity, income, and urbanization rates among global populations, this study matched food consumption data from the FBS with socioeconomic datasets from the FAO statistical database (FAOSTAT) and disease mortality datasets from the Global Burden of Disease (GBD) database. The Global Burden of Disease database is a comprehensive resource organized by global health research institutions to assess and analyze the health impacts of diseases, injuries, and risk factors worldwide and across different regions.

### 3.2. Nutrition Intake Data

To calculate the functional dissimilarity indices and dietary guideline-based functional dissimilarity indices, this study identifies 33 nutrients to define the nutritional functions of foods. These nutrients include total energy, carbohydrates, protein, fat, saturated fatty acids, dietary fiber, cholesterol, total sugars, water, ash, carotene, folate, niacin, pantothenic acid, retinol, riboflavin, thiamine, vitamin A, vitamin B_12_, vitamin B_6_, vitamin C, vitamin D, vitamin E, calcium, copper, iron, magnesium, manganese, phosphorus, potassium, selenium, sodium, and zinc. Calculating nutritional functions requires data on the nutrient conversion coefficients and edible portion coefficients for each food category. Following similar international studies, this study sourced raw data for these coefficients from the USDA National Nutrient Database for Standard Reference, which contains information on 150 nutrients across 8789 food items. To align nutrient intake data with food consumption data, the *USDA* food items were classified into the 14 major food categories defined earlier. After excluding a small number of unclassifiable items, the within-group averages were calculated to derive the nutrient conversion coefficients and edible portion coefficients for each food category. Based on the formula “Nutrient intake = Food consumption × Edible portion coefficient × Nutrient conversion coefficient”, this study compiled per capita intake data for 33 nutrients across 186 countries or regions from 1981 to 2022.

### 3.3. Dietary Guideline Data

To calculate the dietary guideline-based species-neutral indices and dietary guideline-based functional dissimilarity indices, it is also necessary to reference the recommended intake levels for each food category as outlined in dietary guidelines. This study adopts the Global Sustainable Healthy Diet proposed by the EAT-Lancet Commission on Food, Planet, and Health, also known as the EAT-Lancet Diet [31]. This dietary model, which was developed with sustainability in mind, integrates nutritional and health goals for global populations. It emphasizes a diet primarily composed of fruits, vegetables, pulses, whole grains, nuts, and plant-based oils, with moderate consumption of red meat, poultry, dairy, and eggs, and minimal intake of added sugars and saturated fats. The concept of the Global Sustainable Healthy Diet has garnered widespread attention worldwide since its introduction and has been extensively studied and applied by scholars in the fields of food and nutritional health [32,33,34]. The Global Sustainable Healthy Diet provides recommended intake levels and possible ranges for 22 food groups. In this study, these recommendations were converted into 14 food groups to align with the 14 food categories used in this research.

In this study, the adoption of this standard ensures scientific rigor, authority, and comparability. First, the recommended intake principles of the Global Sustainable Healthy Diet are based on extensive evidence from nutritional research and are largely consistent with the dietary guidelines of various countries. For example, common recommendations, such as consuming more fruits, vegetables, and whole grains, limiting processed foods, and avoiding excessive intake of red meat and fats, are shared by the dietary guidelines of most nations [35]. Second, the Global Sustainable Healthy Diet was developed by leading international experts in agriculture, nutrition, environmental sustainability, economics, and policy, which lends it a high level of credibility. Third, employing a globally unified standard allows for horizontal comparisons of dietary diversity across countries, enabling the analysis of regional differences and the identification of international trends. However, relying on a single dietary standard to guide practical applications has its limitations, which will be discussed in detail in Section 6.

Data processing and metric calculations in this study were conducted using Stata software (version: Stata/MP 18.0).

## 4. Results

### 4.1. Characteristics of Global Food Consumption and Nutrition Functional Disparities

Table 2 summarizes the average consumption levels of 14 food categories across the entire sample period and provides a comparison of two distinct time intervals. The results indicate that cereals dominate global dietary patterns, with per capita intake reaching 1501.6 kcal/day, accounting for 53.0% of total caloric intake—far exceeding the combined share of the other 13 food categories. Oils and dairy rank second and third in calorie contribution. Over time, a significant upward trend in per capita food consumption is observed during the first 22 years of the 21st century (2001–2022) compared to the last 20 years of the 20th century (1981–2000). The three food categories with the highest growth rates are poultry, nuts, and vegetables. Total per capita caloric intake rose from 2669.2 kcal/day to 2973.2 kcal/day, with cereals’ share of total energy decreasing by 3.8 percentage points, while sugar’s share increased by 1.3 percentage points.

**Figure 1:**
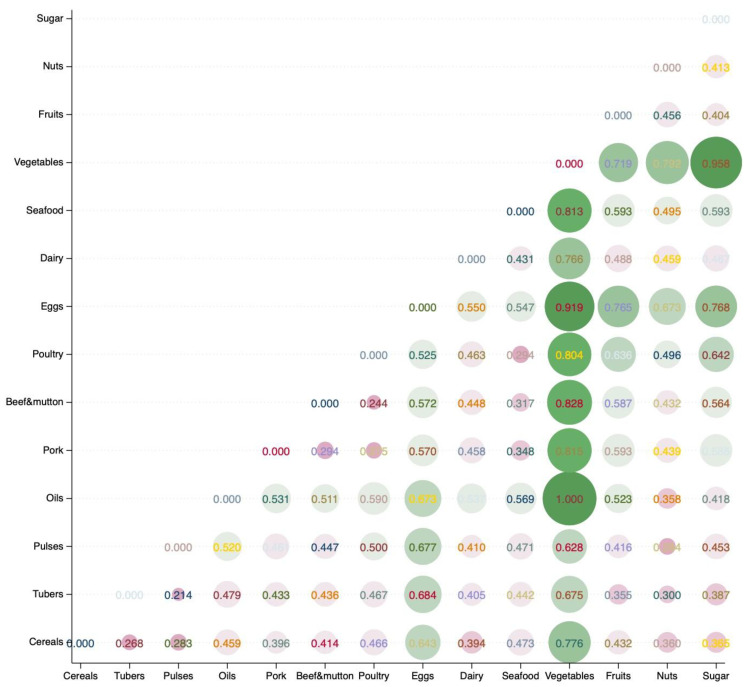
Dissimilarity in nutritional function across food categories. Notes: The values in the figure represent nutrition functional dissimilarity after min–max normalization, with a maximum of 1 and a minimum of 0; larger circles indicate greater functional dissimilarity.

Using the dissimilarity measurement method for nutritional functions discussed earlier, this study applied Euclidean distance to calculate the dissimilarity in nutritional functions among the 14 food categories. The results, presented in Figure 1 after min–max normalization, show that values closer to 1 indicate greater differences in nutritional functions between food categories, while values closer to 0 indicate greater similarity. The analysis reveals significant variation in nutritional functions across different foods. Among the 91 food category combinations (excluding self-to-self comparisons), the average Euclidean distance of nutritional functions was 0.520, with the largest difference observed between vegetables and oils, and the smallest difference observed between tubers and pulses. When ranked by the average distance of their nutritional functions from other food categories, the order from largest to smallest is as follows: vegetables, eggs, oils, sugar, fruits, poultry, seafood, dairy, pork, beef and mutton, nuts, pulses, cereals, tubers. These results suggest that vegetables, eggs, and oils are relatively irreplaceable in terms of nutritional function, while pulses, cereals, and tubers, as staple foods, are more easily substitutable.

### 4.2. Characteristics of Global Dietary Diversity

The correlational validation of the dietary diversity framework reveals systematic alignment with theoretical measurement paradigms (Table 3). When benchmarked against the Shannon Index (*EI*), a widely recognized metric for dietary diversity assessment [36], all indices demonstrated statistically significant associations (*p* < 0.001), confirming framework coherence. The exceptionally high correlation between the Shannon and Simpson indices (Spearman’s rank correlation coefficient r = 0.981) substantiates their classification as species-neutral indices, reflecting equivalent dietary diversity quantification. Moderate associations with *Q_E_* (r = 0.967) and *Q_B_* (r = 0.975) indicate partial convergence with functional dissimilarity indices, although the 3–4% reduction in correlation magnitude relative to Simpson underscores their distinct methodological architecture, which incorporates nutrient-function matrices. Notably, the weak-to-moderate correlations with *D_E_* (r = −0.150), *DQ_E_* (r = 0.550), *DB* (r = 0.453), and *DQ_B_* (r = −0.313) reveal complementary measurement dimensions, verifying the conceptual distinction between species-neutral indices and dietary guideline-based indices. The universal statistical significance (*p* < 0.001) across all pairwise comparisons substantiates the framework’s analytical robustness in differentiating measurement paradigms while maintaining internal consistency within conceptual categories. This empirical validation supports the theoretical proposition that nutritional diversity assessment requires multidimensional quantification through complementary measurement approaches.

Based on the data introduced earlier, this study calculates four categories of dietary diversity indices. Table 4 summarizes the mean, standard deviation (SD), coefficient of variation (CV), skewness, and kurtosis for each index. The results show that the two dietary guideline-based indices have higher means and smaller coefficients of variation compared to the other two indices. Among these, the standardized *D_E_* index has the highest mean (0.914) and the smallest coefficient of variation (0.090), with significantly higher skewness and kurtosis than the other seven indices. In contrast, the *Q_E_* index has the lowest mean (0.559) and the largest coefficient of variation (0.306), along with the lowest skewness and kurtosis. This is because the dietary guideline-based species-neutral indices and dietary guideline-based functional dissimilarity indices measure the relative distance between actual dietary diversity and the recommended diversity based on dietary guidelines. This relative value concept reduces differences among samples and increases centralization around certain values, resulting in indices with higher means, lower dispersion, and more pronounced skewness and kurtosis.

**Figure 2:**
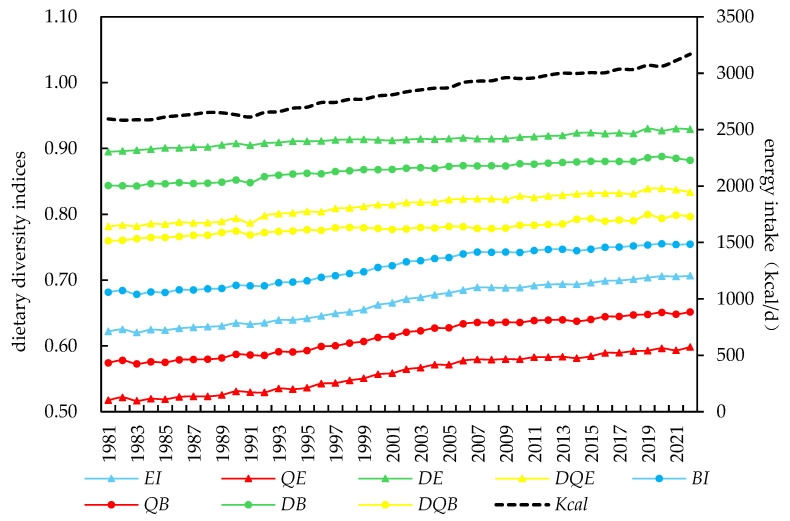
Trends in global dietary diversity: 1981–2022. Notes: 1. To facilitate comparison, the four types of indices were normalized to the range [0, 1] using min–max standardization within the sample range, and negative indices were converted into positive ones through a complement transformation method. 2. Each color represents a type of dietary diversity index: the blue curve represents species-neutral indices, the red curve represents functional dissimilarity indices, the green curve represents dietary guideline-based species-neutral indices, and the yellow curve represents dietary guideline-based functional dissimilarity indices.

Figure 2 illustrates the global trends in dietary diversity from 1981 to 2022. The eight dietary diversity indices show a clear temporal trend: global dietary diversity has steadily increased over time. The variety of foods available has expanded, evenness in food distribution has improved, the gap between nutritional functions and dietary guideline recommendations has narrowed, and overall dietary quality has continuously improved. These positive changes have been driven by a combination of factors, including the refinement of food supply systems, the deepening of international food trade, advancements in food processing and storage technologies, rising household incomes, and a growing awareness of healthy eating habits [12,37,38]. Additionally, sustained efforts by governments and international organizations to address food security and nutrition issues, supported by funding and policy initiatives, have significantly contributed to the improvement of dietary diversity worldwide.

Among the indices, dietary guideline-based species-neutral indices (*D_E_* and *D_B_*) and dietary guideline-based functional dissimilarity indices (*DQ_E_* and *DQ_B_*) exhibit higher absolute levels, whereas species-neutral indices (*EI* and *BI*) and functional dissimilarity indices (*Q_E_* and *Q_B_*) demonstrate faster growth rates. This observation aligns with the statistical findings in Table 2. However, Figure 2 also reveals the significant impact of the COVID-19 pandemic on global dietary diversity. While per capita calorie intake increased noticeably in 2020 and 2021 compared to pre-pandemic levels, changes in dietary quality, as reflected by different indices, varied: (1) *EI* and *BI* indices show that the upward trend in dietary quality remained unaffected by the pandemic, (2) *DQ_E_* and *D_B_* indices indicate a continuous decline in dietary diversity starting in 2020, (3) *D_E_* and *DQ_B_* indices show a decline in dietary quality in 2020, partial recovery in 2021, and another drop in 2022, and (4) *Q_E_* and *Q_B_* indices exhibit the opposite trend, with a significant improvement in 2020, a decline in 2021, and another rise in 2022. These contrasting patterns reflect the complex and multifaceted effects of the pandemic on global dietary diversity and food systems.

### 4.3. Characteristics of Global Dietary Diversity in Different Dietary Patterns

This study categorizes global diets into 13 regionally representative dietary patterns, taking into account the dietary habits and cultural differences of residents in various regions. It is important to note that global dietary patterns are highly complex, diverse, and distinctive. The 13 dietary patterns discussed here are merely typical representatives on a global scale; they are widely recognized, highly representative, and exhibit significant differences from one another. This does not suggest that other dietary patterns are nonexistent or unimportant. In fact, dietary patterns do not have strictly defined national boundaries, and many countries may embody multiple dietary patterns, making the mapping relationship relatively intricate. The representative countries for each dietary pattern were determined by the authors based on empirical research and relevant information on geography, culture, and diet. These associations are not absolute and do not imply a strict relationship of inclusion or exclusion. The aim of comparing the dietary diversity differences among typical dietary patterns is to uncover regularities and draw meaningful conclusions. The representative countries and core characteristics of each dietary pattern are summarized in Table A1. Asia includes four typical dietary patterns: East Asian Dietary Pattern (E-ASIA), Southeast Asian Dietary Pattern (SE-ASIA), South Asian Dietary Pattern (S-ASIA), and Central West Asian Dietary Pattern (CW-ASIA). Europe is represented by two patterns: Northeast European Dietary Pattern (NE-EURO) and Mediterranean Dietary Pattern (MEDI), the latter spanning parts of Western and Southern Europe, as well as parts of the Middle East and North Africa. Africa is divided into the East South African Dietary Pattern (ES-AFRI) and Central West African Dietary Pattern (CW-AFRI). The Americas include four patterns: North American Dietary Pattern (N-AMER), Latin Caribbean Dietary Pattern (LACA), Andean Dietary Pattern (ANDE), and South American Dietary Pattern (S-AMER). Lastly, Oceania is represented by a single dietary pattern: Oceanian Dietary Pattern (OCEA).

Dietary diversity exhibited significant spatial-temporal heterogeneity across geographical regions (13 regionally representative dietary patterns) and years (Table 5). Geographical variations were systematically analyzed through Bartlett’s test for variance homogeneity and ANOVA, while temporal patterns were investigated using mixed-effects models that incorporated both fixed annual trends and random regional effects. The universal significance of all test statistics (*p* < 0.001) provides robust evidence for three fundamental conclusions: (1) substantial heteroscedasticity exists across dietary patterns for all dietary indices, (2) both inter-regional disparities and longitudinal changes demonstrate statistically meaningful magnitudes, and (3) regional clusters display distinct temporal trajectories in diversity evolution. The analytical findings underscore the necessity of conducting dietary diversity assessments that systematically differentiate both geographical variations in dietary patterns and temporal evolutionary characteristics.

**Figure 3:**
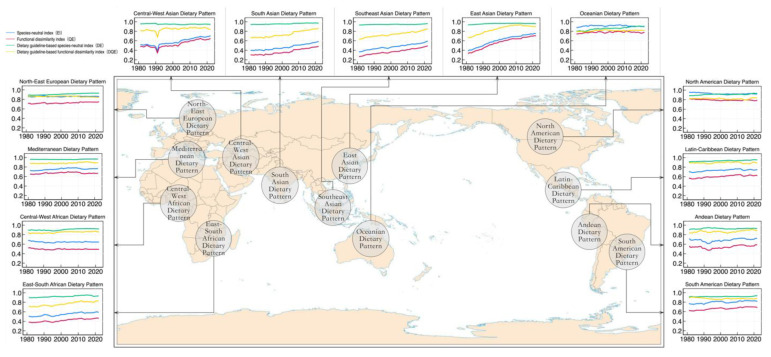
Geographic distribution of global typical dietary patterns and trends in dietary diversity (1981–2022). Notes: 1. To facilitate comparison, the four types of indices were normalized to the range [0, 1] using min–max standardization within the sample range, and negative indices were converted into positive ones through a complement transformation method. 2. Each color represents a type of dietary diversity index: the blue curve represents species-neutral indices, the red curve represents functional dissimilarity indices, the green curve represents dietary guideline-based species-neutral indices, and the yellow curve represents dietary guideline-based functional dissimilarity indices.

Figure 3 illustrates the geographical distribution of these dietary patterns and the trends in dietary diversity from 1981 to 2022 using four *EI*-series indices as representatives (due to space limitations, the calculation results of the *BI*-series dietary diversity indices are not displayed). Over time, dietary diversity has improved across nearly all dietary patterns. The two indices based on dietary guidelines exhibit lower absolute values but greater increases, corroborating the global average results shown in Figure 2. Several key findings emerge: (1) Despite the overall improvement in global dietary diversity, certain dietary patterns experienced slight declines in specific indices, such as the *EI* and *Q_E_* indices for the Central West African Dietary Pattern, the *Q_E_* index for the North American Dietary Pattern, the *D_E_* index for the Central West Asian Dietary Pattern, and the *DQ_E_* index for both the Northeast European and South American Dietary Patterns. (2) Developing countries, particularly those in Asia, exhibit faster growth in dietary diversity across all four indices, with the indices showing a clear convergence trend. (3) Traditional Western dietary patterns, such as those in Northeast Europe, North America, and Oceania, show slower growth in dietary diversity, with their four indices already converging at relatively stable levels. In contrast to traditional Western nations that maintain high and stable dietary diversity, developing countries are experiencing rapid improvement and convergence in dietary diversity. This divergence is driven by accelerated household income growth, urbanization, infrastructure investment, and optimization of food system support policies. Enhanced food supply systems have increased food accessibility, while relaxed budget constraints and targeted policy interventions have collectively facilitated the fulfillment of diversified dietary demands. (4) Other regional dietary patterns, while also stabilizing, still display significant differences in the levels of the four indices, indicating that convergence has not yet been achieved.

**Figure 4:**
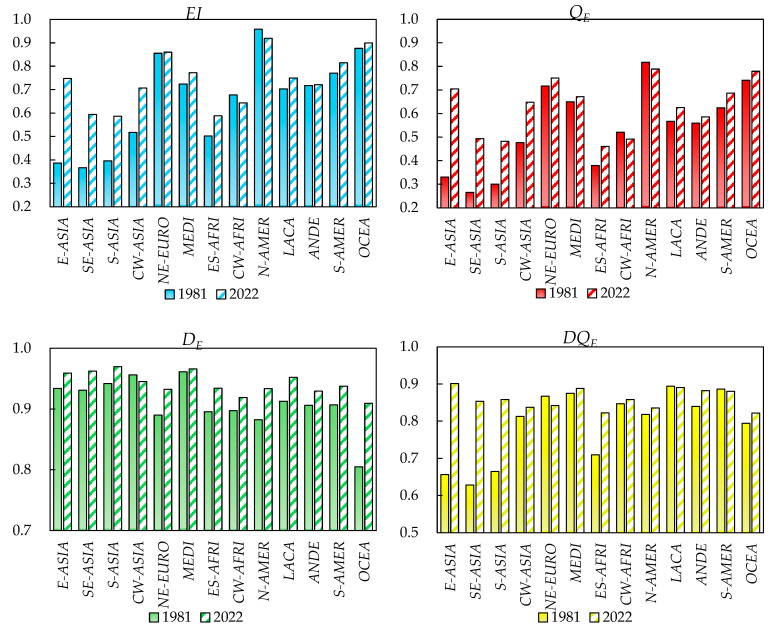
Dietary diversity of global typical dietary patterns (1981 and 2022).

Figure 4 highlights the comparison of dietary diversity across different patterns in 1981 and 2022. Using the *EI* index—which does not account for dietary guidelines or the nutritional functionality of foods—as an example, the results show that in 1981, the top three dietary patterns with the highest *EI* values were the North American Dietary Pattern (0.958), the Oceanian Dietary Pattern (0.876), and the Northeast European Dietary Pattern (0.855). By 2022, while the dietary diversity of the North American Dietary Pattern had slightly declined—narrowing its gap with other patterns—the top three rankings remained the same, with *EI* values of 0.919, 0.899, and 0.860, respectively. Over the 40-year period, the East Asian Dietary Pattern showed the highest growth in *EI*, increasing from 0.387 in 1981 to 0.747 in 2022, with its ranking rising from 12th to 7th. The other three Asian dietary patterns also exhibited relatively high increases in *EI* values.

Using the *DQ_E_* index—which incorporates both dietary guidelines and the nutritional functionality of foods—as another example, the results reveal a shift in rankings. In 1981, the top three dietary patterns with the highest *DQ_E_* values were the Latin Caribbean Dietary Pattern, the South American Dietary Pattern, and the Mediterranean Dietary Pattern. By 2022, the top three shifted to the East Asian Dietary Pattern, the Latin Caribbean Dietary Pattern, and the Mediterranean Dietary Pattern. The East Asian Dietary Pattern demonstrated a significant leap, rising from 12th to 1st place. This improvement is attributed to its plant-based nature, high vegetable consumption (nutritionally distant from other food groups), rich food variety, and balanced diet, making it closely aligned with global healthy dietary standards. In contrast, dietary patterns with high *EI* values, such as the North American, Oceanian, and Northeast European Dietary Patterns, ranked lower in the *DQ_E_* index due to their heavy reliance on meat and dairy, which deviate more significantly from diversity and health standards.

### 4.4. Relationships Among Dietary Diversity, Income, and Urbanization Rate

Income and urbanization rate are key drivers that economists consider when analyzing the transformation and upgrading of food consumption patterns. According to Bennett’s Law and extensive empirical research, dietary diversity generally increases with rising income levels. At the same time, due to significant differences in food consumption patterns between urban and rural populations, the increase in urbanization driven by population migration is also regarded as a critical factor influencing food consumption demand [39]. Using the *EI* and *DQ_E_* indices as examples, this study explores the relationships among dietary diversity, per capita income, and the urbanization rate.

**Figure 5:**
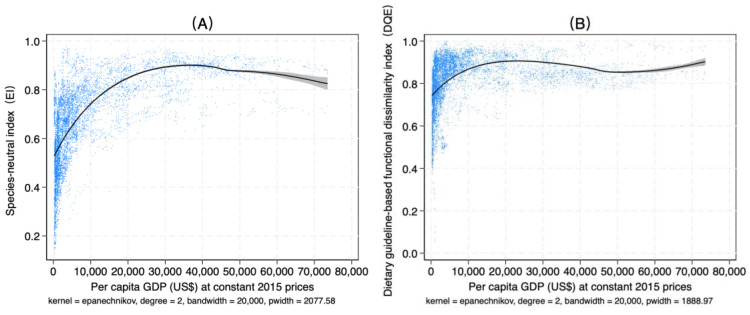
Relationship between dietary diversity and per capita income. Notes: (**A**) Relationship between *EI* and per capita income. (**B**) Relationship between *DQ_E_* and per capita income. A local polynomial regression fitting was used to explore the nonlinear relationship between dietary diversity and income. The black line represents the fitted curve, while the gray area indicates the 95% confidence interval.

Figure 5 illustrates the relationship between dietary diversity and income levels across countries. On a global scale, dietary diversity follows three distinct developmental stages as per capita income increases. The first is the diversity enhancement stage, in which the evenness of food categories steadily improves, and the gap between dietary nutritional functionality and recommended dietary standards gradually narrows. This trend aligns with Bennett’s Law, suggesting that income growth promotes dietary diversity. Additionally, as the proportion of high-value food consumption rises, dietary quality also improves. However, the impact of income on dietary diversity and quality exhibits diminishing marginal returns, with the peak of dietary diversity occurring at an income range of approximately USD 20,000 to USD 40,000. The second stage is the diversity decline stage, where dietary diversity begins to decrease after reaching its peak. This decline is driven by relaxed income constraints, which amplify preference-driven food consumption. Consequently, the evenness of food categories diminishes, leading to health concerns such as obesity and a deviation in nutritional functionality from recommended dietary standards. The third stage is the dietary quality enhancement stage, during which the *DQ_E_* index rises again. With rising income levels, people’s dietary habits and health awareness continue to improve. At the same time, advancements in information dissemination and food technology empower individuals to proactively and precisely adjust their dietary patterns based on nutritional knowledge. By strategically incorporating functional foods into their diets, they can effectively address limitations in food variety or deficiencies in high-value foods, thereby improving overall dietary quality.

**Figure 6:**
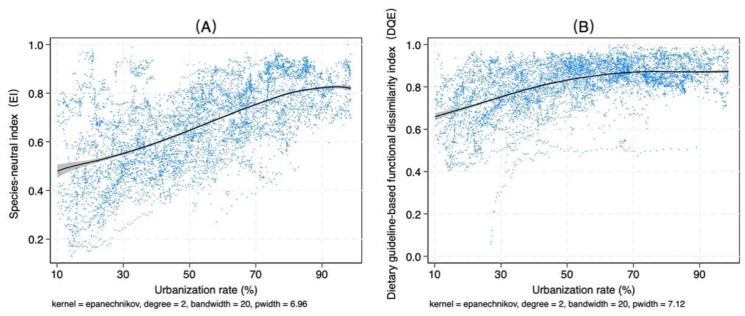
Relationship between dietary diversity and urbanization rate. Notes: (**A**) Relationship between *EI* and urbanization rate. (**B**) Relationship between *DQ_E_* and urbanization rate. A local polynomial regression fitting was used to explore the nonlinear relationship between dietary diversity and the urbanization rate. The black line represents the fitted curve, while the gray area indicates the 95% confidence interval.

Figure 6 shows the relationship between dietary diversity and urbanization rate across countries. As urbanization increases, the evenness of food categories improves, the gap between dietary nutritional functionality and recommended dietary standards narrows, and dietary diversity rises. However, after reaching a certain level of urbanization, dietary diversity peaks and then gradually declines. Notably, the changes in the *EI* and *DQ_E_* indices with urbanization display distinct characteristics. First, the peak of the *EI* index occurs later, at an urbanization rate of over 90% (Figure 6A), whereas the *DQ_E_* index peaks earlier, at approximately 70% urbanization (Figure 6B). Second, the *DQ_E_* index exhibits diminishing marginal returns during its growth phase, while the *EI* index shows no significant diminishing returns during its prolonged growth phase. It only begins to exhibit diminishing returns as it nears its peak.

## 5. Discussion

The key findings of this study are further discussed along three dimensions. First, future research on dietary diversity should carefully select one or more of the four index categories based on specific research objectives, placing greater emphasis on the nutritional functional differences among foods and clarifying the relationship between dietary diversity and dietary health. Conceptually, species-neutral indices measure the average rarity of food intake; functional dissimilarity indices measure the weighted average rarity of foods based on their nutritional functions; dietary guideline-based species-neutral indices assess the gap in average rarity of food categories between actual diets and reference dietary standards; and dietary guideline-based functional dissimilarity indices measure the gap in the weighted average rarity of nutritional functions between actual diets and reference dietary standards. Among these, species-neutral diversity indices, such as the Shannon Entropy Index (*EI*) and Simpson Index (*BI*), are the most widely utilized in existing research [36]. Incorporating nutritional functional differences among foods highlights the disparity in dietary diversity, emphasizing the relationship between dietary patterns and the functional contributions of nutrients [29,30]. This approach enables the identification of differences in dietary diversity patterns with similar richness and evenness, thereby enhancing the comprehensiveness of dietary diversity measurement. Moreover, integrating dietary guidelines into these indices establishes a monotonic relationship between dietary diversity and dietary health: the closer the diet adheres to the recommended standards, the higher the level of dietary health. This integration makes dietary diversity indices a valuable component of dietary health assessments.

Second, there is a pressing need to enhance the inclusivity and precision of healthy dietary recommendations, placing greater emphasis on dietary diversity in both its promotion and measurement. This study finds that dietary diversity, measured against dietary guideline recommendations, is a critical dimension for evaluating dietary health [16]. As demonstrated earlier, using the EAT-Lancet Diet as a benchmark for healthy eating provides authoritative guidance and ensures global comparability, facilitating academic research and pattern exploration [31,34]. However, global dietary patterns are diverse and unique, shaped by variations in resource endowments, dietary habits, and levels of economic development [40,41]. Relying solely on a single dietary guideline standard to inform practice has its limitations. It is worth noting that approximately 100 countries or regions worldwide have developed national dietary guidelines tailored to their local populations. According to FAO statistics, more than 100 countries and regions worldwide have developed and published food-based dietary guidelines in various forms. For more details, please refer to: https://www.fao.org/nutrition/education/food-dietary-guidelines/home/en/, accessed on 5 December 2024. Yet, many of these guidelines fall short of achieving their comprehensive objectives [35,42]. Some focus exclusively on nutritional health, overlooking the need to synergize economic, cultural, and health factors. Furthermore, most guidelines emphasize dietary diversity only in terms of food variety, neglecting aspects such as evenness and disparity. Therefore, dietary guidelines should be optimized to account for nutritional health, environmental sustainability, economic affordability, and cultural acceptability, balancing the interests of diverse population groups. Improvements in dietary diversity recommendations should emphasize the importance of balanced food categories and the functional contributions of nutrition.

Third, an analysis of dietary diversity trends across global dietary patterns reveals that developing countries and regions must remain vigilant and take timely measures to prevent future declines in dietary diversity and health outcomes [4,5]. The findings indicate that, over the past four decades, rising dietary diversity levels in developing countries and regions, such as Asia, sub-Saharan Africa, Central America, and South America, have significantly contributed to improving global dietary health. However, lessons from developed countries suggest that dietary health often follows a trajectory of initial improvement followed by subsequent decline as economic development and urbanization progress. This phenomenon is largely attributable to the misalignment between the welfare goals of food consumption and health objectives. In the early stages of socioeconomic development, transitions such as “from insufficient to sufficient food”, “from poor-quality to high-quality diets”, and “from monotonous to diverse diets” typically signify overall improvements in dietary health. However, at higher levels of development, further transitions, such as “better”, “more diverse”, and “more enjoyable” diets, do not necessarily equate to healthier eating. Moreover, once developed countries enter the phase of dietary health decline, correcting the misalignment between welfare and health goals often proves to be a prolonged and costly process, requiring considerable health and fiscal resources [34,41]. To avoid similar pitfalls, developing countries should promote the healthy transformation of their food processing industries, allocate policies and funding to the production of healthier foods, optimize and disseminate healthy dietary patterns, and draw on the strengths of diverse global dietary models. Additionally, enhancing the communication of healthy dietary standards and diet-related disease risks within dietary guidelines is critical for mitigating the risks of diet-related illnesses.

This study acknowledges several limitations. First, as the research measures dietary diversity at a macro level, the analysis relies on secondary data from authoritative sources such as the FAO. While these datasets are extensively utilized and demonstrate established reliability, future investigations would benefit from incorporating primary micro-level dietary data to validate the findings. Second, the adoption of the EAT-Lancet Diet as the nutritional benchmark introduces potential measurement biases. Although this standard integrates both health and sustainability considerations, it may not fully align with the dietary priorities of low- and middle-income countries, where food security and basic nutritional adequacy remain predominant concerns. Third, the exploration of relationships between dietary diversity and socioeconomic drivers, as well as the identification of optimal diversity thresholds, requires deeper investigation. However, given this study’s primary focus on methodological framework development and index quantification, these aspects will be rigorously addressed through model-based approaches in subsequent research.

## 6. Conclusions

Dietary diversity is an essential component of a healthy diet and plays a vital role in both academic research and policymaking. Against the backdrop of global food security governance and food system transformation, enhancing dietary diversity has emerged as a critical lever for promoting healthier dietary habits and facilitating lifestyle improvements. While existing studies offer numerous methods and indices to measure dietary diversity, a lack of conceptual clarity often limits their interpretability. This ambiguity obscures the precise meanings of these indices and blurs the boundaries between them. In this study, we first elucidated the conceptual origins and definitions of dietary diversity measurement. Building on this foundation, we proposed a new measurement framework that classifies dietary diversity indices along two dimensions: whether the index accounts for the nutritional functional differences among foods and whether it incorporates dietary guidelines. Additionally, we developed several new indices based on the Shannon Entropy Index and Simpson Index to address the limitations of existing measurement systems. These indices enable a more comprehensive evaluation of dietary diversity. Consequently, dietary diversity indices were categorized into four types: species-neutral indices, functional dissimilarity indices, dietary guideline-based species-neutral indices, and dietary guideline-based functional dissimilarity indices.

Using per capita consumption data for 14 food categories across countries from 1981 to 2022, this study applied eight indices to calculate global dietary diversity and systematically analyzed its variation across 13 dietary patterns. Furthermore, the relationship between dietary diversity, income levels, and urbanization rates was explored from a global perspective. The findings indicate that most indices effectively capture the upward trends in global dietary diversity and dietary quality. Regional differences in dietary diversity reflect the interplay between dietary patterns and health, highlighting the combined influence of food resource endowments, cultural and dietary habits, and levels of socioeconomic development. More specifically, the non-linear relationships observed between dietary diversity indices, income, and urbanization rates align closely with Bennett’s Law and findings from extensive empirical studies. These results underscore the robust informational value of the dietary diversity indices developed in this study.

## Figures and Tables

**Table 1:** Four types of dietary diversity indices.

		Considering the Nutrition Functional Dissimilarity	
		NO	YES
Considering the dietary guidelines		(1) Species-neutral indices	(2) Functional dissimilarity indices
	NO	$\mathrm{EI}=-\sum_{i}^{S}p_{i}\ln\left(p_{i}\right)$	$Q_{B}=\sum_{i}^{S}\sum_{j}^{S}d_{ij}p_{i}p_{j}$
		$\mathrm{BI}=1-\sum_{i}^{S}p_{i}^{2}$	$Q_{E}=-\sum_{i}^{S}p_{i}\ln\left(1-\sum_{j\neq i}^{S}d_{ij}p_{j}\right)$
		(3) Dietary guideline-based species-neutral indices	(4) Dietary guideline-based functional dissimilarity indices
	YES	$D_{E}=\sum_{i}^{S}p_{i}\left(\ln\left(p_{i}\right)-\ln\left(q_{i}\right)\right)$	$DQ_{E}=\sum_{i}^{S}p_{i}\left|\ln\left(1-\sum_{j\neq i}^{S}d_{ij}p_{j}\right)-\ln\left(1-\sum_{j\neq i}^{S}d_{ij}q_{j}\right)\right|$
		$D_{B}=\sum_{i}^{S}p_{i}\left|p_{i}-q_{i}\right|$	$DQ_{B}=\sum_{i}^{S}\sum_{j}^{S}d_{ij}p_{i}\left|p_{j}-q_{j}\right|$

**Table 2:** Per capita food consumption of global residents (1981–2022).

Foods	1981–2022				1981–2000				2001–2022			
	Quantity (kg/year)		Energy (kcal/d)		Quantity (kg/year)		Energy (kcal/d)		Quantity (kg/year)		Energy (kcal/d)	
	Mean	SD	Mean	SD	Mean	SD	Mean	SD	Mean	SD	Mean	SD
Cereals	146.8	54.3	1501.6	555.6	143.9	56.9	1471.9	581.8	149.2	52.0	1526.1	531.9
Tubers	56.3	63.8	121.9	137.9	54.5	71.3	118.0	154.2	57.8	56.8	125.1	122.8
Pulses	6.8	6.4	18.4	17.4	6.7	6.3	18.2	17.1	6.8	6.5	18.6	17.6
Oils	12.3	6.8	302.9	167.7	11.2	6.7	274.9	165.5	13.2	6.7	326.0	165.9
Pork	11.7	15.0	44.0	56.1	10.2	14.6	38.2	54.9	13.0	15.1	48.8	56.6
Beef and mutton	14.7	13.6	55.7	51.5	15.3	15.2	57.9	57.5	14.2	12.1	53.9	45.8
Poultry	15.5	15.3	40.9	40.3	11.0	12.0	29.0	31.5	19.3	16.7	50.8	44.0
Eggs	6.3	5.3	22.9	19.2	5.6	5.0	20.3	18.2	6.9	5.4	25.1	19.7
Dairy	88.1	73.9	161.6	135.6	79.7	70.4	146.1	129.1	95.1	76.0	174.3	139.4
Seafood	15.0	15.3	42.1	43.0	14.2	15.6	40.0	43.7	15.6	15.1	43.8	42.4
Vegetables	84.1	64.9	62.8	48.5	72.1	58.7	53.9	43.9	93.9	67.9	70.2	50.8
Fruits	80.4	56.5	110.6	77.7	73.4	54.1	101.0	74.4	86.1	57.7	118.5	79.4
Nuts	5.1	5.7	59.2	66.0	3.9	4.7	45.2	54.3	6.1	6.2	70.8	72.3
Sugar	35.0	21.5	291.1	179.2	30.6	18.6	254.7	155.2	38.6	23.0	321.2	191.7
Energy			2835.7				2669.2				2973.2	
Obs	104,314				47,166				57,148			

Data source: Food Balance Sheets (FBS) compiled by the FAO.

**Table 3:** Spearman’s rank correlation coefficient (r) of dietary diversity indices.

	*EI*	*Q_E_*	*D_E_*	*DQ_E_*	*BI*	*Q_B_*	*D_B_*	*DQ_B_*
*EI*	1							
*Q_E_*	0.967 ***	1						
*D_E_*	−0.150 ***	−0.147 ***	1					
*DQ_E_*	0.550 ***	0.521 ***	0.515 ***	1				
*BI*	0.981 ***	0.953 ***	−0.238 ***	0.527 ***	1			
*Q_B_*	0.975 ***	0.997 ***	−0.168 ***	0.530 ***	0.972 ***	1		
*D_B_*	0.453 ***	0.435 ***	0.575 ***	0.950 ***	0.445 ***	0.447 ***	1	
*DQ_B_*	−0.313 ***	−0.347 ***	0.826 ***	0.451 ***	−0.365 ***	−0.357 ***	0.449 ***	1

Notes: *** *p* < 0.001.

**Table 4:** Statistical analysis of dietary diversity indices.

	(1) Species-Neutral Indices		(2) Functional Dissimilarity Indices		(3) Dietary Guideline-Based Species-Neutral Indices		(4) Dietary Guideline-Based Functional Dissimilarity Indices	
	*EI*	*BI*	*Q_E_*	*Q_B_*	*D_E_*	*D_B_*	*DQ_E_*	*DQ_B_*
Mean	0.666	0.721	0.559	0.615	0.914	0.867	0.813	0.779
SD	0.173	0.175	0.171	0.171	0.082	0.093	0.117	0.118
CV	0.260	0.243	0.306	0.278	0.090	0.107	0.144	0.151
Skewness	0.484	0.834	0.379	0.547	3.505	1.861	1.362	2.359
Kurtosis	2.599	3.132	2.524	2.724	21.047	9.308	5.261	11.248
	7409	7409	7409	7409	7409	7409	7409	7409

**Table 5:** Geographical variation and temporal trend tests.

Dietary Diversity Indices	Geographical Variation Tests				Temporal Trend Tests			
	Bartlett’s Equal Variances Test		ANOVA		Fixed Effects		Random Effects	
	χ^2^	*p*-Value	F	*p*-Value	Coef	*p*-Value	Inter-Regional Variance	*p*-Value
*EI*	608.338	0.000	446.640	0.000	0.0024	0.000	0.0242	0.000
*QE*	574.357	0.000	363.580	0.000	0.0026	0.000	0.0204	0.000
*DE*	310.340	0.000	163.880	0.000	0.0008	0.000	0.0008	0.000
*DQE*	566.338	0.000	74.950	0.000	0.0018	0.000	0.0027	0.000

## Data Availability

The original contributions presented in the study are included in the article, further inquiries can be directed to the corresponding author.

## Abbreviations

The following abbreviations are used in this manuscript:

E-ASIA	East Asian Dietary Pattern
SE-ASIA	Southeast Asian Dietary Pattern
S-ASIA	South Asian Dietary Pattern
CW-ASIA	Central West Asian Dietary Pattern
NE-EURO	Northeast European Dietary Pattern
MEDI	Mediterranean Dietary Pattern
ES-AFRI	East South African Dietary Pattern
CW-AFRI	Central West African Dietary Pattern
N-AMER	North American Dietary Pattern
LACA	Latin Caribbean Dietary Pattern
ANDE	Andean Dietary Pattern
S-AMER	South American Dietary Pattern
OCEA	Oceanian Dietary Pattern

## Appendix A

**Table A1:** Representative countries and typical characteristics of different dietary patterns.

Dietary Pattern	Representative Countries	Diet Composition	Key Features
East Asian Dietary Pattern	China, Japan, South Korea	Primarily based on rice, noodles, and wheat, with protein from pork, chicken, fish, tofu, and eggs, alongside a variety of vegetables and light oils.	Diverse, vegetable-rich, low-fat.
South Asian Dietary Pattern	India, Pakistan	Focuses on rice, lentils, and flatbreads, with protein from pulses, chicken, mutton, fish, and dairy, heavily spiced with turmeric, cumin, and other spices.	Spice-heavy, vegetarian-influenced.
Southeast Asian Dietary Pattern	Thailand, Vietnam, Malaysia, Indonesia	Centered on rice and noodles, with protein from chicken, fish, and shrimp, seasoned with coconut, herbs, and chili, offering a mix of sweet, sour, and spicy flavors.	Sweet, sour, and spicy flavors.
Central West Asian Dietary Pattern	Kazakhstan, Uzbekistan, Iran	Relies on bread, rice, and lentils as staples, with protein from mutton, beef, chicken, fish, and yogurt, heavily flavored with spices and herbs.	Rich in spices and dairy.
Northeast European Dietary Pattern	Sweden, Germany, Russia, Poland	Features potatoes and rye bread as staples, with protein from pork, beef, fish, cheese, and dairy, alongside simple vegetable dishes.	High-protein, simple cooking.
Mediterranean Dietary Pattern	Italy, Greece, Spain, Turkey	Emphasizes whole-grain bread, pasta, and vegetables, with protein from fish, chicken, pulses, and nuts, seasoned with olive oil and herbs.	Olive oil- and fish-centric.
North American Dietary Pattern	USA, Canada	Includes bread, potatoes, and corn as staples, with high consumption of beef, chicken, pork, and processed foods like fast food and snacks.	High in processed foods.
Latin Caribbean Dietary Pattern	Mexico, Cuba, Colombia	Based on tortillas, rice, and beans, with protein from chicken, pork, and fish, heavily spiced with chili and accompanied by tropical fruits.	Corn and bean-based, spicy.
South American Dietary Pattern	Brazil, Argentina	Combines rice and black beans with protein from beef, chicken, and pork, often prepared as barbecue and served with coffee or mate tea.	Beef-heavy, barbecue culture.
Andean Dietary Pattern	Peru, Ecuador	Relies on quinoa, roots and tubers, and corn as staples, with protein from guinea pig, beef, chicken, and fish, complemented by regional herbs and spices.	Higher consumption of Roots and Tubers
Central West African Dietary Pattern	Nigeria, Ghana, Senegal	Built around cassava, yams, and plantains, with protein from fish, chicken, and mutton, seasoned with palm oil and spicy herbs.	Cassava-based, spicy.
East South African Dietary Pattern	Ethiopia, Uganda, South Africa	Primarily uses maize, cassava, and rice as staples, with protein from beef, mutton, chicken, fish, and dairy, seasoned with spices and butter.	Dairy and meat emphasis.
Oceanian Dietary Pattern	Australia, New Zealand	Features bread, potatoes, and rice as staples, with protein from beef, lamb, chicken, fish, and dairy, influenced by Western and Asian cuisines.	Meat-heavy, Western influence.
